# On the automaticity of relational stimulus processing: The (extrinsic) relational Simon task

**DOI:** 10.1371/journal.pone.0186606

**Published:** 2017-10-26

**Authors:** Adriaan Spruyt, Jan De Houwer

**Affiliations:** Department of Experimental-Clinical and Health Psychology, Ghent University, Ghent, Belgium; University College London, UNITED KINGDOM

## Abstract

We introduce the (extrinsic) relational Simon task as a tool for capturing automatic relational stimulus processing. In three experiments, participants responded to a perceptual relation between two stimuli. Results showed that participants were faster and more accurate to respond when the (task-irrelevant) conceptual relation between these stimuli was compatible (rather than incompatible) with the (extrinsic) relational meaning of the required responses. This effect was replicated irrespective of the type of stimulus materials used, irrespective of the similarity between the relational information that was task-relevant and the relational information that was task-irrelevant, and irrespective of the complexity of the task-irrelevant relational information. Our findings add to a growing body of evidence showing that relational stimulus processing can occur under conditions of automaticity.

## Introduction

Life presents itself as a bombardment of stimuli and experiences. One way to handle this massive quantity of stimulation is *selection*. It is well-known that humans are equipped with the ability to selectively attend to specific types of stimuli [1] or even to specific stimulus dimensions [2, 3]. A second way to handle massive stimulus input is *automation*, that is, the ability to process stimulus information very quickly, efficiently, outside awareness, and/or in the absence of an (explicit) processing goal [4].

Research on automatic stimulus processing has focused almost exclusively on the (semantic) analysis of a single stimulus. In the semantic priming literature, for example, literally hundreds of studies have been published concerning the automatic extraction of meaning from a single word (e.g., [5]). In the real world, however, a successful exchange between an organism and its environment typically involves a comparative integration of different sources of information. Even the simple act of buying a hamburger requires a comparison of different response options as a function of the stimulus environment and both short-term and long-term goals. It is therefore quite surprising that relatively little research has focused on the possibility that the analysis of the precise way in which two (or more) stimuli relate to each can occur under automaticity conditions (hereafter referred to as *automatic relational stimulus processing*). At least to some extent, this state of affairs seems to have resulted from the predominant view that automatic processes must be associative in nature whereas complex relational stimulus processing must be non-automatic in nature (e.g., [6]). There are, however, no a priori theoretical reasons to map the quality of a given process (i.e., a comparison between two stimuli vs. the analysis of a single stimulus) onto the automaticity conditions under which this process can operate [7, 8]. In other words, it is a matter of empirical research to describe what kind of processes can take place under different automaticity conditions. As soon as one adopts this approach, it becomes clear that, in principle, processes operating under automaticity conditions might be much more complex than previously assumed. In fact, several reports of automatic relational stimulus processing have already appeared in the literature, although the reported effects were not always described as such.

As a first example, consider the findings reported by Heider and colleagues [9]. In a series of four sequential priming studies, they used line drawings of large and small objects as target stimuli (e.g., a truck vs. a spoon) and asked participants to judge the size of these objects relative to either a small reference object (i.e., a football) or a large reference object (i.e., a car). Crucially, the prime set included line drawings of objects that were larger than the small reference object but smaller than the large reference object (i.e., a bike). Results showed that the impact of these ‘intermediate’ primes on target responding was dependent upon the magnitude of the reference object. When the reference object was small, the intermediate prime stimuli facilitated responses towards targets larger than the reference object relative to responses towards targets smaller than the reference object. In contrast, if the reference object was large, the same set of intermediate prime stimuli facilitated responses towards targets smaller than the reference object relative to responses towards targets larger than the reference object. Importantly, the primes were completely irrelevant for the target task. Accordingly, whereas the requirement to engage in a relational target task was probably an important prerequisite for the occurrence of the relational priming effect, the findings obtained by Heider and colleagues [9] demonstrated that the relational meaning of stimuli can be processed even in the absence of the explicit requirement to do so (i.e., goal-dependent unintentionality, see [4]; for related findings, see [10–16]).

The relational priming studies by Heider and colleagues [9] are somewhat limited, however, in the sense that the same two reference objects were used throughout the entire experimental session. As a result, the representation of the reference objects must have been highly activated in working memory, which may be an important prerequisite for automatic relational stimulus processing to take place. Research suggests, however, that this is probably not the case. Perhaps the best example comes from studies using an experimental task design that we will refer to as the Relational Stroop Task (hereafter referred to as *RST*). As an example, consider the findings reported by Paivio [17]. In Experiment 2, Paivio [17] presented participants with pairs of pictures (48 pairs in total) or printed names (also 48 pairs in total) of objects and animals differing in real-life size. Crucially, the physical size of the individual stimuli (i.e., the image size or font size) was manipulated such that the physical size relation and the conceptual size relation were either congruent (e.g., ‘lamp–ZEBRA’) or incongruent (e.g., ‘LAMP–zebra’). When asked to judge whether the conceptually larger member of each pair was presented on the left or the right, participants were clearly faster to respond on congruent pairs than on incongruent pairs, at least for the picture pairs (i.e., no effects were obtained with the word pairs). That is, similar to a classic Stroop task, performance was better when the relevant stimulus dimension (i.e., the conceptual size relation) and the irrelevant stimulus dimension (i.e., the physical size relation) pointed to the same response as compared to different responses (see [18], p. 237–238).

Importantly, this RST effect occurred despite the fact that participants were to ignore the (task-irrelevant) physical size relation and therefore supports the idea that relational processing of physical size can take place automatically [4]. Moreover, given the use of a large number of stimulus pairs, the findings of Paivio [17] suggest that automatic relational stimulus processing can occur even for stimuli that are not actively stored in working memory. It may be noted, however, that Paivio [17] himself did not interpret his findings in terms or automatic relational stimulus processing. Instead, he seemed to endorse an explanation in terms of “internal analog representations that contain relative size information along with other attributes of the objects” (p. 646). It may also be noted that we readily replicated the RST effect in two unpublished studies, just like several other authors (e.g., [19–22]). As is the case for all data reported in this article, the raw data of these (unpublished) studies are available at https://figshare.com. They can be accessed directly via the following links: https://doi.org/10.6084/m9.figshare.5005940.v1 (for study 1, *N* = 21) and https://doi.org/10.6084/m9.figshare.5005985.v1 (for RST study 2, *N* = 34).

Interestingly, a special variant of the RST was used by Klauer and Musch [23]. They presented participants with pairs of words and manipulated both the evaluative meaning of these words (i.e., positive vs. negative) and an orthogonal, non-evaluative stimulus dimension (i.e., location, color, letter case, or grammatical category, in Experiments 5, 6, 7, and 8 respectively). Results showed that participants were faster to indicate that two words were similar in terms of a non-evaluative stimulus dimension (e.g., letter case) when these words were also similar in terms of their evaluative meaning (e.g., ‘love—pretty’ or ‘HATE—UGLY’) as compared to when these words had a different evaluative meaning (e.g., ‘love—ugly’ or ‘HATE—PRETTY’). Likewise, participants were faster to indicate that two words were dissimilar in terms of a non-evaluative stimulus dimension when these words were also dissimilar in terms of their evaluative meaning (e.g., ‘love—UGLY’ or ‘HATE—pretty’) as compared to when these words had the same evaluative meaning (e.g., ‘LOVE—pretty’ or ‘hate—UGLY’). In other words, task performance was best when task-irrelevant and the task-relevant relational stimulus information promoted the same response (for reviews of related findings, see [24, 25]). In structural terms, this setup is identical to the RST used by Paivio [17], except for the fact that nature of the relational information under study was different (i.e., a simple similarity relation vs. a more complex size relation, [18]). Similar findings in the perceptual domain were reported, amongst others, by Proctor and colleagues [26].

As explained above, the findings obtained with the RST are important because they suggest that automatic relational stimulus processing can occur even if the stimuli that enter the comparison process are not stored in working memory. Still, the usefulness of the RST as a tool to study automatic relational stimulus processing is limited. By definition, the RST requires dimensional overlap [27] between a task-relevant and a task-irrelevant (relational) stimulus dimension. Crucially, it is also a requirement that one of these relational dimensions can be manipulated via perceptual stimulus properties. As a result, the use of the RST is limited to the measurement of automatic relational stimulus processing in the domain of concrete, physical relations. For example, the ability to process conceptual size relations under automaticity conditions can be readily examined by asking participants to respond on the basis of the physical size relation between stimuli (and vice versa) [17]. It is unclear, however, how one could adjust the RST to capture automatic appraisals of abstract relations. It would be very difficult (if not impossible), for example, to construct an RST that allows for the measurement of the automatic appraisal of causal relations. In fact, even the simple automatic appraisal that one food option (e.g., a fresh salad) is healthier than another food option (e.g., a greasy hamburger) would be difficult to capture using the RST. Of course, one might note that the RST variant in which participants respond on the basis of the similarity/dissimilarity of stimuli [23] is quite flexible because it can be used to capture automatic similarity/dissimilarity appraisals across of a wide range of relational domains. However, by definition, this version of the RST does not allow one to probe the automatic appraisal of relational information more complex than simple similarity relations.

These problems can be sidestepped, however, by adopting a Simon-like response task [28]. In the classic Simon task, the compatibility of a required response and a task-irrelevant stimulus dimension varies over trials [18]. Craft and Simon [29], for example, asked participants to respond with a left or right key press based on the color of red and green stimuli presented on the left or the right of a screen. Results showed that participants were faster and more accurate to respond when the spatial position of the required response matched the location of the stimulus than when the spatial position of the required response and the stimulus were different. In sum, performance in a Simon-like response task is better when the required response is compatible with the task-irrelevant stimulus dimension as compared to when the required response is incompatible with the task-irrelevant stimulus dimension. In the past, this basic principle was already used successfully to study automatic evaluative stimulus processing [30] and (non-evaluative) semantic stimulus processing [31]. In the studies by De Houwer and Eelen [30], for example, participants were found to be faster in pronouncing positive and negative words based on a non-evaluative feature of (single) words (i.e., grammatical category) when the evaluative meaning of these words matched rather than mismatched the evaluative meaning of the (correct) response. Likewise, we suspected that the execution of a relational response on the basis of a task-relevant relation between two stimuli might be affected by a task-irrelevant relation between these stimuli, provided of course that there is dimensional overlap between the (relational) response set and the task-irrelevant relation under study.

The approach developed here was in part inspired by Moors and De Houwer [13] who used a Simon procedure to study the automatic appraisal of dominance and submissiveness. In their experiments, participants were presented with complex visual scenes showing the same two persons in different submissive/dominant relationships (e.g., an army officer shouting at a soldier). Each of the two persons acted as a dominant person on some trials and as a submissive person on other trials. Orthogonal to the manipulation of the nature of the interpersonal relationship, each person appeared equally often on the right and the left side of the visual scene and participants were asked to pronounce the words ‘dominant’ and ‘submissive’ based on the location of one target actor. Results showed that participants were faster to respond when the relational meaning of the responses matched the actual relational status of the target actor, despite the fact that relational stimulus information was in fact task-irrelevant. In principle, one can apply this logic to any type of relational information, although the authors themselves did not foresee this broad generalization. Therefore, the main aim of the present research was to demonstrate the generality of this relational Simon effect.

In Experiment 1, we presented participants with pairs of synonyms and antonyms and asked them to pronounce the words “synonym” and “antonym” depending on the match/mismatch in letter case. That is, in line with the general make-up of a Simon task, we manipulated the compatibility of two relational responses on the one hand and a task-irrelevant conceptual relation between two words on the other hand (i.e., the relational Simon task). Assuming that participants would process the (task-irrelevant) conceptual relation between the words automatically, we hypothesized that task performance would be better if the required relational response was compatible rather than incompatible with the conceptual relation. In Experiment 2, we replicated Experiment 1 using non-verbal responses. More specifically, we asked participants to judge a perceptual relation between two words on one subset of trials (i.e., same vs. different letter case) and to judge a conceptual relation between two words on the remaining trials (i.e., synonyms vs. antonyms). Crucially, both types of relatedness were manipulated orthogonally. Based on the assumption that participants would engage in an automatic appraisal of task-irrelevant relational information, we expected task performance to be better when the task-relevant and the task-irrelevant information promoted the same response as compared to a different response (i.e., the extrinsic relational Simon task). Finally, in Experiment 3, we sought to demonstrate that one could use the (extrinsic) relational Simon task to capture the automatic appraisal of complex comparative stimulus information.

## Experiment 1

### Ethics statement

All studies reported in this manuscript were approved by the Ethics Committee of the Faculty of Psychology and Educational Sciences of Ghent University (approval number 2015/40). All participants gave (written) informed consent prior to participation.

### Method

#### Participants

Participants were 20 students at Ghent University (1 man, 19 women). They all received course credit in exchange for their participation. All participants were Dutch speakers and had normal or corrected-to-normal vision.

#### Materials

Stimuli were eight word pairs consisting of synonyms and eight word pairs consisting of antonyms. Synonym and antonyms pairs were matched so that both words of one synonym pair (e.g., STRONG—POWERFUL) were both antonyms of the two words of another synonym pair (e.g., WEAK—FAINT). The individual words of the matched synonym pairs were used to create two antonym pairs (e.g., STRONG—WEAK and POWERFUL—FAINT). Independently of their meaning, words were presented either in uppercase of lowercase letters.

Stimuli were presented in white (font Tahoma, font size 28, RGB 255, 255, 255) against the black background of a 27-inch computer monitor (100 Hz, screen resolution 1024 x 768, RGB 0, 0, 0). An Affect 4.0 program [32] controlled the presentation of the stimuli as well as the registration of the response latencies. An external voice key that was connected to the parallel port of the computer was used to register the response latencies.

#### Procedure

Participants were tested individually in a darkened room. Each trial started with a 500-ms presentation of a (white) fixation stimulus (i.e., ‘+’, Arial, font size 28, RGB 255, 255, 255) in the center of the computer screen. Next, 500 ms after the offset of the fixation cross, a word pair was presented. One word was presented above the location of the fixation cross (i.e., 38 pixels, counting from upper side of the word) and one word was presented below the location of the fixation cross (i.e., 38 pixels, counting from the lower side of the word). Participants pronounced the word ‘SYNONYM’ when both words were presented in the same letter case. Word pairs presented in a different letter case required the pronunciation of the word ‘ANTONYM’. Instructions emphasized the importance of responding as quickly as possible. As soon as the voice key detected a sound, both words were cleared from the computer screen. The experimenter coded the accuracy of the verbal responses as well as the accuracy of the triggering of the voice key by pressing one of four keys on the computer keyboard. A feedback message was presented for 2000 ms if participants pronounced the wrong word (i.e., ‘INCORRECT!!!’, Arial, font size 28, RGB 255, 0, 0). The same feedback message was presented if participants gave an invalid response (e.g., pronouncing one of the words, saying ‘euh’, etc.). If something went wrong with the triggering of the voice key, the word ‘MICROPHONE!!!’ was presented for 2000 ms (Arial, font size 28, RGB 128, 128, 255). Valid responses that were both correct and registered accurately by the voice key were not followed by a feedback message. The inter-trial interval varied randomly between 500 ms and 1500 ms.

Each participant completed two blocks of 128 trials each. Within each block, each word pair was presented 8 times and each individual word was presented exactly 16 times. Within each block, the following experimental variables were balanced: the location of the individual words (i.e., above or below the location of the fixation stimulus), the letter case of the individual words (i.e., uppercase vs. lowercase), the perceptual relationship between the two words (i.e., same vs. different letter case), and the relationship between the two words in terms of their meaning (i.e., synonyms vs. antonyms).

### Results

The raw data of this experiment are available at https://doi.org/10.6084/m9.figshare.5005508.v1. At the group level, there were no outliers in terms of overall response time and/or error rate. Mean response latencies were computed after the exclusion of trials on which an incorrect response (3.79%), an invalid response (1.56%), a far-out value (2.13%), or an incorrect triggering of the voice key (4.69%) was registered. Far-out values were defined as values that deviated more than 2.5 standard deviations from the mean of an individual participant in a particular cell of the design [33]. There were no outliers in terms of the overall error rate or the overall mean response latency.

The mean response latency observed on compatible trials was 730 ms (*SD* = 44 ms). The mean response latency observed on incompatible trials was 750 ms (*SD* = 68 ms). The difference between both conditions (i.e., the relational Simon effect) was reliable, *t*(19) = 3.31, *p* < .005, *d* = .74. No effect emerged in the error data, *t* < 1. The mean error rate on compatible trials was 3.83% (*SD* = 2.30%). The mean error rate on incompatible trials was 3.75% (*SD* = 2.37%).

### Discussion

As predicted, the relational Simon effect reached significance. Participants were faster to pronounce a relational word in response to a perceptual relation between two words when the conceptual relation between these words was compatible with the required response as compared to when it was incompatible with the required response. As such, our findings provide initial evidence for the potential of the relational Simon task as a new tool for studying relational stimulus processing. The question now arises whether the effects observed in this task qualify as ‘automatic’. It is widely acknowledged that automaticity is an umbrella term for a variety of different automaticity features (e.g., unintentional, uncontrollable, unconscious, and fast) that may or may not co-occur [4, 34–36]. Accordingly, to evaluate whether automatic processes drive a given effect, a systematic analysis of each of these automaticity features is required.

Reassuringly, there are good reasons to argue that the relational Simon effect observed in Experiment 1 is indeed characterized by several automaticity features. First, because the conceptual relational information was task-irrelevant throughout the entire experimental procedure, it seems unlikely that participants adopted the (conscious) intention to process it. In fact, even the opposite may have been true. Remember that the conceptual relational information promoted the incorrect response on 50% of the trials (i.e., 50/50 compatible and incompatible trials). Participants thus had a good reason to avoid processing the conceptual relational between the words of each stimulus pair. The observation that the relational Simon effect did reach significance thus suggests that this effect was driven (at least to some extent) by uncontrollable processes. Second, it is important to realize that for a relational Simon effect to occur, participants need to have processed six chunks of information: a task-relevant and a task-irrelevant feature of two different stimuli (i.e., 4 chunks in total) as well as two types of relational information (i.e., 2 chunks in total). In contrast, for a standard (non-relational) Simon effect to occur, participants need to have processed just two chunks of information: a task-relevant and a task-irrelevant feature of a single stimulus. Even though a mean response latency of 740 ms is somewhat long in comparison with standard (non-relational) Simon tasks (e.g., [37], but see [30]), it can be thus concluded that the underlying processes of the relational Simon effect are fast and efficient.

In sum, our findings demonstrate that one can use the relational Simon task to capture the automatic appraisal of complex abstract relations between pairs of stimuli. In principle, this task is now available to study any type of conceptual relational stimulus processing. For example, to examine the automatic appraisal of causality, one might think of constructing a task in which participants pronounce the words “cause” or “consequence” as a function of a (task-relevant) physical relation between two words.

## Experiment 2

The relational Simon task as implemented in Experiment 1 has a practical limitation. By definition, a Simon task requires dimensional overlap between the response set and some task-irrelevant stimulus dimension. Accordingly, for the present research purposes (i.e., automatic relational stimulus processing), it is a requirement that the response set includes response options that are tied to the relational information under investigation. This requirement is easy to meet using verbal responses. In Experiment 1, for example, we simply asked participants to pronounce the words “synonym” and “antonym”, that is, two words that acquired a strong relational meaning because of the learning history of an individual. The use of verbal (relational) responses makes it difficult, however, to run large-scale or online studies because, typically, the presence of an experimenter is required to operate a voice key.

To resolve this issue, in Experiment 2, we adapted the experimental procedure used in Experiment 1 so that participants were required, on a subset of trials, to respond with a left or right keypress based on the conceptual relation between two words (i.e., synonyms vs. antonyms). On the remaining trials, participants used the same set of keys to judge a perceptual similarity relation (i.e., the match/mismatch in letter case). Crucially, we manipulated the conceptual and the perceptual relational dimension independently from each other. As a result, the (correct) response was either compatible or incompatible with the task-irrelevant relation on every trial. For two reasons, we anticipated that participants would be faster and/or more accurate to respond on compatible trials as compared to incompatible trials. First, each of the two responses might acquire an extrinsic relational meaning [38] that either matches or mismatches with the task-irrelevant relational dimension. According to this interpretation, the anticipated compatibility effect would result from (dimensional) overlap between the response set(s) and the irrelevant stimulus dimension(s) [27]. Alternatively, and perhaps more parsimoniously, one could also argue that it is simply easier to execute a response in a task-switch design if the relevant and the irrelevant task promote the same as compared to a different response (i.e., the so-called task-rule congruency effect, see [39]). According to this interpretation, the anticipated compatibility effect would result from overlap between two (otherwise unrelated) response sets (i.e., sometimes referred to as ‘RR compatibility’ [40]). We will return to this issue in the discussion section of Experiment 2.

### Method

#### Participants

Participants were 33 students at Ghent University (4 men, 29 women). They all received course credit in exchange for their participation. All participants were Dutch speakers and had normal or corrected-to-normal vision.

#### Materials

All materials were identical to those used in Experiment 1, except for the use of a (standard) computer keyboard instead of an external voice key to register the responses.

#### Procedure

The experimental procedure of Experiment 2 was almost identical to the experimental procedure of Experiment 1. Participants again completed two blocks of 128 trials each (i.e., 256 trials in total). Within each block, each word pair was presented eight times and each individual word was presented exactly 16 times. As was the case in Experiment 1, the location of the individual words, the letter case of the individual words, the perceptual relationship between the two words (i.e., same vs. different letter case), and the relationship between the two words in terms of their meaning (i.e., synonyms vs. antonyms) were perfectly balanced. In addition, orthogonal to these factors, we manipulated the color of the word pairs. On exactly 50% of the trials, the two words appeared in a white font (Tahoma, font size 28, RGB 255, 255, 255; hereafter referred to as *white trials*). On the remaining trials, the two words appeared in a blue font (Tahoma, font size 28, RGB 0, 255, 255; hereafter referred to as *colored trials*). Participants switched between two experimental tasks, depending on the color of the word pairs. For the colored trials, we asked participants to press a right key if both words were synonyms and the left key if both words were antonyms. For the white words, we asked participants to press the right key if both words appeared in the same letter case and the left key if both words appeared in a different letter case. As was the case in Experiment 1, an error message was presented for 2000 ms if participants made an error (i.e., ‘INCORRECT!!!’, Arial, font size 28, RGB 255, 0, 0) and the inter-trial interval varied randomly between 500 ms and 1500 ms.

### Results

The raw data of this experiment are available at https://doi.org/10.6084/m9.figshare.5011655.v1. Mean response latencies were again computed after the exclusion of trials on which an incorrect response (12.20%) or a far-out value (2.46%) was registered. Far-out values were defined as values that deviated more than 2.5 standard deviations from the mean of an individual participant in a particular cell of the design [33]. The data of one participant were excluded from the analyses because his/her overall response latency (i.e., 2312 ms) exceeded our cutoff criterion of 2.5 standard deviations above the grand mean (*M* = 1472 ms, *SD* = 317 ms; threshold = 2265 ms). The data of two other participants were excluded because of excessive error rates (i.e., 40.23% and 33.59%) in comparison to the complete sample (*M* = 12.20%, *SD* = 7.81%; threshold = 31.74%). In sum, the analyses reported below are based on a final sample of 30 participants. None of the results below were contingent upon the exclusion of participants. Detailed descriptive statistics are provided in Table 1.

**Table 1:** Mean response latencies (in ms) and mean error rates (in percentages) as a function of trial type and compatibility in Experiment 2 (*SE* in parentheses).

	White trials		Colored trials	
Dependent variable	Compatible	Incompatible	Compatible	Incompatible
Reaction time data	1192 (38)	1219 (42)	1742 (62)	1794 (59)
Error data	5.83 (0.76)	13.13 (1.37)	8.49 (1.14)	14.69 (1.62)

The reaction time data were analyzed by means of a 2 (trial type: white trials vs. colored trials) × 2 (compatibility: compatible trials vs. incompatible trials) repeated measures ANOVA. The main effect of trial type was highly reliable: *F*(1, 29) = 205.69, *p* < .001, *d* = 2.62. Participants were much slower to respond on colored trials (*M* = 1768 ms, *SE* = 38.79 ms) as compared to white trials (*M* = 1206 ms, *SE* = 58.80 ms), suggesting that the synonym/antonym judgment task was much more difficult than the size judgment task. More importantly, the only other effect that reached significance was the main effect of compatibility, *F*(1, 29) = 4.23, *p* < .05, *d* = .38. As expected, participants were faster to respond on compatible trials (*M* = 1467 ms, *SE* = 47.16 ms) as compared to incompatible trials (*M* = 1506 ms, *SE* = 46.36 ms). There was no statistical evidence whatsoever that the compatibility effect was different for white trials and colored trials, *F* < 1.

The same set of tests was performed for the error data. In line with the reaction time data, the critical main effect of compatibility was highly reliable, *F*(1, 29) = 53.28, *p* < .001, *d* = 1.33. Participants responded more accurately on compatible trials (*M* = 7.16%, *SE* = 1.09%) as compared to incompatible trials (*M* = 13.91%, *SE* = 1.59%). The (theoretically less important) main effect of trial type failed to reach significance, *F*(1, 29) = 2.34, *p* = .14, *d* = .28. There was again no evidence for an interaction between the two factors, *F* < 1.

### Discussion

On each of a series of trials, participants judged either a conceptual or a perceptual relation between two words using the same response keys. Results showed that task performance was better when the two relational tasks promoted the same response as compared to when the two relational tasks promoted a different response, a phenomenon that we refer to as the extrinsic relational Simon effect. At the methodological level, Experiment 2 thus introduces a novel task to study relational stimulus processing. At the mental-process level, the question arises whether the effects observed in this new task qualify as ‘automatic’.

For several reasons, we believe that the answer to this question is affirmative. First, based on the observation that participants were apparently unable to cancel out the influence of task-irrelevant relational information, one could argue that the processes driving our effects were (at least to some degree) difficult to control (for a similar argument in the context of a non-relational Simon effect, see [41]). In addition, the occurrence of a reliable extrinsic relational Simon effect implies that relational processing can take place even if participants are actively engaged in performing another relational task. One can thus conclude that relational stimulus processing as measured by the extrinsic relational Simon task is efficient. What about the speed criterion? At first sight, the observation that participants needed more than a full second to respond (on average) seems incompatible with the idea that fast-acting processes were at play. It is important to realize, however, that the experimental procedures used in this experiment were quite complex. In fact, for the extrinsic relational Simon effect to come about, participants needed to have processed seven chunks of information: a task-relevant and a task-irrelevant feature of two experimental stimuli (i.e., 4 chunks in total), two types of relational information (i.e., 2 chunks in total), and a task cue (i.e., 1 chunk). Relatively speaking then, even though the overall response speed was somewhat elevated relative to more traditional (i.e., non-relational) Simon studies, one could argue that fast-acting processes were driving our effects. Some caution is in order, however, when evaluating the unintentionality criterion of automaticity. Unlike to what was the case in Experiment 1, participants were never able to anticipate the nature of the upcoming response task. Hence, one might argue that (at least some) participants processed the task-irrelevant dimension in an intentional manner (on at least a subset some trials). For example, participants could have treated one task as the default task and the second task as the exception task that had to be executed only if necessary. Alternatively, participants could have been inclined, on each new trial, to repeat the task just performed during the preceding trial. Both scenarios would result in an intentional processing of the task-irrelevant stimulus dimension on a specific subset of trials (i.e., trials on which participants needed to perform the non-dominant task or trials on which participants needed to switch between tasks, respectively). Accordingly, if the occurrence of the extrinsic relational Simon effect would be limited to these trials, it would be difficult to entertain the idea that unintentional processes were driving our effects. Reassuringly, however, additional analyses seem to rule out these scenarios. First, using the asymmetric switch cost as an index of task dominance [42, 43], we found no statistical evidence that the extrinsic relational Simon effect was reliable only if the task-irrelevant stimulus dimension matched the dominant task-set (*F*s < 1). Second, the extrinsic relational Simon effect did reach significance on both task-switch trials and repetition trials, at least in the error data. Taken together then, it seems safe to conclude the processes underlying the extrinsic relational Simon effect are automatic in the sense of uncontrollable, efficient, fast, and unintentional. Nevertheless, it might be worthwhile to conduct additional studies to rule out the operation of intentional strategies even more firmly. We will return to this issue in the General Discussion.

Although the processes underlying the extrinsic relational Simon task seem to qualify as automatic, the exact nature of these processes still needs to be determined. There are at least two possibilities. First, one could argue that each of the two responses acquired a double relational meaning (i.e., one for each task) that either matched or mismatched with the relational information that was task-irrelevant [38]. According to this viewpoint, the observed compatibility effect resulted from (dimensional) overlap between the response set(s) and the irrelevant stimulus dimension(s) [27]. As an alternative (but not mutually exclusive) interpretation, one may also argue that the observed compatibility effect resulted from overlap between two (otherwise unrelated) response sets. The latter explanation can account for to the current findings because participants switched between two (relational) tasks (i.e., the letter-case comparison task and the synonym/antonym judgment task) based on a perceptual feature that was itself unrelated to each of these relational tasks (i.e., the color of the words, white vs. blue). This approach diverges, for example, from the extrinsic affective Simon task [44] in which participants are asked to switch between a color judgment task (blue vs. green) and a valence judgment task based on the color of a series of words (white vs. not white). Moreover, we manipulated both the conceptual and the perceptual relation between the words of each stimulus pair independently of the nature of the response task. As a result, the compatibility between the required response and the task-relevant relational dimension varied across all trials, that is, both in the letter-case comparison task and the synonym/antonym judgment task. This make-up underscores that the extrinsic relational Simon effect can be seen as an instance of the much broader class of task-rule congruency effects (e.g., [39]; for a related discussion, see [45]).

This viewpoint has no influence, however, on the validity of our conclusions. In fact, there was no statistical evidence that the magnitude of the compatibility effect was different on white trials as compared to colored trials (*F* < 1, both in the error data and the response latency data). Moreover, the compatibility effect reached significance both on the white trials and the colored trials (at least in the error data). We can thus safely conclude that participants were able to process both the conceptual and the perceptual stimulus dimension under automaticity conditions and that one can exploit the extrinsic relational Simon effect as a cognitive marker of (automatic) relational stimulus processing. Importantly, the use of arbitrary keypresses as responses makes the current procedure highly versatile. It thus seems possible to study any type of (automatic) relational stimulus processing using a task-switch design in which participants press arbitrary keys in response to different types of relational stimulus information.

## Experiment 3

The results of Experiments 1 and 2 suggest that one can use the (extrinsic) relational Simon task to capture (automatic) relational stimulus processing. Still, the generalizability of these findings is somewhat limited, for two reasons. First, in Experiment 2, participants switched between two relational tasks that were highly similar to each other. Judging whether two words are synonyms is, in a sense, a similarity judgment task, just like the letter-case comparison task. So, participants switched between a conceptual and a perceptual similarity judgment task, which may have been a prerequisite for the (extrinsic) relational Simon effect to occur. Second, even if a high degree of similarity between the two relational tasks was not a necessary precondition for the effect to occur, the relational information under study was relatively simple. Whereas existing tools already allow for a measurement of (automatic) similarity judgments (e.g., [23]), it is a unique strength of the (extrinsic) relational Simon task that it can be readily adjusted to allow for the measurement of complex rank-order judgments.

Accordingly, to demonstrate this versatility of the (extrinsic) relational Simon task, we conducted a final study in which participants switched between two unrelated relational tasks, one of which required rank-order judgments. In Experiment 3, we presented participants with pairs of digits either in the same color or in a different color. On one subset of trials (indicated by the presentation of two digits in white), participants indicated as quickly as possible whether the largest digit appeared on the left or the right side of the computer screen. On the remaining trials (indicated by the presentation of digits in red, green, blue or yellow), a simple color similarity judgment was required. We thus switched back to a traditional extrinsic Simon approach in the sense that an orthogonal manipulation of the two relational dimensions was restricted to one subset of (diagnostic) trials. Despite the fact that the two relational tasks were quite dissimilar, we expected participants to respond faster and more accurately on the color judgment trials if the (task-irrelevant) size relation between the digits promoted the same versus a different response.

### Method

#### Participants

Participants were 25 students at Ghent University. Due to a programming error, demographic data (i.e., age and gender) were not saved by the computer program. All participants received course credit in exchange for their participation, were Dutch speakers, and had normal or corrected-to-normal vision.

#### Materials

Stimuli were numbers between 0 and 9, presented in white (font Tahoma, font size 28, RGB 255, 255, 255) against the black background of a 27-inch computer monitor (100 Hz, screen resolution 1024 x 768, RGB 0, 0, 0). Responses were registered using a standard, AZERTY computer keyboard. All other aspects, the materials used were identical to those used in Experiment 2.

#### Procedure

Participants were tested individually in a darkened room and completed two blocks of 186 trials (i.e., 372 trials in total). On each trial, two different numbers were presented. One number was presented to the left of the fixation cross (i.e., 51 pixels, counting from the left side of the number) and one number was presented to the right of the fixation cross (i.e., 51 pixels, counting from the right side of the number). On 90 trials, both numbers were presented in white (RGB 0, 0, 0; hereafter referred to as *white trials*). On the remaining trials (hereafter referred to as *color trials*), the individual numbers were presented in red (RGB 255, 0, 0), blue (RGB 0, 0, 255), green (0, 255, 0), or yellow (RGB 255, 255, 0). For the white trials, participants were asked to indicate as quickly as possible whether the largest number was presented on the left or the right side of the computer screen by pressing either the left or the right response key, respectively. For the colored trials, participants were asked to decide as quickly as possible whether the two numbers were presented in the same color or in a different color. Response assignments for the color trials were counterbalanced. One group of participants (*n* = 13) pressed the right and the left response key to indicate a color match and a color mismatch, respectively. A second group of participants (*n* = 12) pressed the right response key to indicate a color mismatch and the left key to indicate a color match.

On exactly 50% of the color trials, the two numbers were presented in the same color. On the remaining color trials, the two numbers were presented in a different color. Within the subset of color trials, each color was presented equally often and the frequency of different combinations of colors was perfectly balanced. Likewise, the location of the largest number of each pair (left or right) was perfectly balanced, within both the subset of colored trials and the subset of white trials. Importantly, within the subset of colored trials, the location of the largest number was manipulated independently of the color match (or mismatch) between the two numbers. In sum, on the critical color trials, the proportion of compatible and incompatible trials was exactly 50%. Within the subset of white trials, (a) each number was presented equally often on each location (i.e., 9 times) and (b) each possible combination of numbers was presented exactly once. Within the subset of the color trials, the individual numbers were sampled semi-randomly, with the restrictions specified above.

Each trial started with a 500-ms presentation of a (white) fixation stimulus (i.e., ‘+’, Arial, font size 28, RGB 255, 255, 255) in the center of the computer screen. Next, 500 ms after the offset of the fixation cross, a pair of numbers was presented. A feedback message was presented for 2000 ms if participants pressed the wrong response key (i.e., ‘INCORRECT!!!’, Arial, font size 28, RGB 255, 0, 0). The inter-trial interval varied randomly between 500 ms and 1500 ms.

### Results

The raw data of this experiment are available at https://doi.org/10.6084/m9.figshare.5008973.v1. The data of one participant were excluded from the analyses because his/her overall mean response latency (i.e., 1083 ms) exceeded our cutoff criterion of 2.5 standard deviations above the grand mean (*M* = 729 ms, *SD* = 113 ms; threshold = 1011 ms). Likewise, the data of one participant were excluded because of an exceptionally high error rate (i.e., 32.81%) in comparison to the complete sample (*M* = 10.52% ms, *SD* = 7.72%; threshold = 29.81%). Analyses were restricted to the 192 (critical) color trials. Mean response latencies were computed after the exclusion of trials on which an incorrect response (9.24%) or a far-out value (2.33%) was registered. Far-out values were again defined as values that deviated more than 2.5 standard deviations from the mean of an individual participant in a particular cell of the design (see [33]). Detailed descriptive statistics are provided in Table 2.

**Table 2:** Mean response latencies (in ms) and mean error rates (in percentages) as a function of response assignment and compatibility in Experiment 3 (*SE* in parentheses).

	Left key = color match		Right key = color match	
Dependent variable	Compatible	Incompatible	Compatible	Incompatible
Reaction time data	704 (26.12)	742 (27.27)	695 (27.28)	731 (28.59)
Error data	6.33 (1.13)	11.89 (3.35)	4.36 (1.18)	14.39 (3.50)

The reaction time data were analyzed by means of a 2 (response assignment) × 2 (compatibility) repeated measures ANOVA. The only effect that reached significance was the main effect of compatibility (i.e., the extrinsic relational Simon effect), *F*(1, 21) = 20.17, *p* < .001, *d* = .96. As predicted, participants were faster to respond on compatible trials (*M* = 699 ms, *SE* = 18.18 ms) as compared to incompatible trials (*M* = 737 ms, *SE* = 19.72 ms). An analysis of the error data revealed the same pattern of effects. The main effect of compatibility was reliable, *F*(1, 21) = 9.98, *p* < .001, *d* = .65, and did not depend on the nature of the response assignment, *F* < 1. Participants made fewer errors on compatible trials (*M* = 5.35%, *SE* = 0.82%) as compared to incompatible trials (*M* = 13.14%, *SE* = 2.42%).

### Discussion

The extrinsic relational Simon effect again reached significance, both in the response latency data and the error data. It may be noted that Experiment 3 was a replication of an earlier study (*N* = 36) that was almost identical to the experiment reported here, with the following exceptions. First, the response assignments for the color trials was not counterbalanced (i.e., all participants pressed the right and the left response key to indicate a color match and a color mismatch, respectively). Second, the experimental stimuli ranged between 1 and 10 (instead of between 0 and 9). Third, there was just one block of 186 trials (instead of two blocks of 186 trials each). The results of this experiment mimic the results of the experiment reported here. Both in the response latency data, *t*(35) = 5.22, *p* < .001, *d* = .87, and the error data, *t*(35) = 6.47, *p* < .001, *d* < 1.08, a clear-cut compatibility effect emerged. The raw data of this experiment are available at https://doi.org/10.6084/m9.figshare.5008919.v1.

Despite the complexity of the experimental setup, the mean response latencies observed in Experiment 3 were roughly in the same range as those observed in Experiment 1 (i.e., well below one second). This observation confirms our earlier assessment that relational stimulus processing as measured by the extrinsic relational Simon task can be fast and efficient. In line with Experiment 2, additional analyses also confirmed that the extrinsic relational Simon effect (a) was reliable on both task-switch trials and task-repetition trials and (b) did not depend on task dominance as indexed by the asymmetric switch costs (*F*s < 1). These observations are again in line with the idea that the occurrence of the extrinsic relational Simon effect is not critically dependent upon the explicit intention to process the task-irrelevant stimulus dimension. Finally, one can again argue that the processes underlying the extrinsic relational Simon effect are (at least to some extent) uncontrollable because, apparently, participants were unable to overcome the influence of task-irrelevant relational stimulus information. In sum, the findings of Experiment 3 add further weight to the idea that automatic processes are driving the extrinsic relational Simon effect.

More importantly, the findings of Experiment 3 also extend the findings of Experiment 1 and 2, in two ways. First, given the use of digits rather than words, our findings suggest that automatic relational stimulus processing generalizes across different types of stimulus materials. Second, participants switched between two response tasks that were qualitatively very different. Whereas (unqualified) similarity judgments were needed on one subset of trials, rank-order judgments were needed on the remaining trials. The observation that the extrinsic relational Simon effect replicated under these conditions thus implies that automatic relational stimulus processing generalizes to complex comparative relations and that one can use the extrinsic relational Simon task to capture this phenomenon.

One may note, however, that some caution is in order when interpreting the findings of Experiment 3. Remember that participants performed an explicit size-comparison task on the white trials. The experimental stimuli on these trials consisted of digits ranging from 0 to 9 and large digits were, by definition, most often the largest digit of a digit pair. Small digits, in contrast, were most often the smallest digit of a digit pair. It could thus be argued that participants may have been biased to select the response option that corresponded with the location of large digits, that is, irrespective of an online assessment of the size relation between the two digits (for a related discussion, see [17]). If this were true, the extrinsic relational Simon effect reported here would not be indicative of automatic relational stimulus processing at all. To rule out this rivaling interpretation, we reanalyzed our data using two specific subsets of trials. A first subset included trials with digits ranging between 4 and 6. In other words, the digits used on this subset of trials appeared both as the smallest and as the largest digit of a digit pair on a relatively large number of trials. It was thus unlikely that the response bias described above was operative on these trials. The second subset consisted of trials with digits smaller than 4 and larger than 6. The largest digit of these pairs was thus the largest digit on most of the trials, thereby promoting the response bias described above. If our findings were simply a by-product of a response bias, the extrinsic relational Simon effect should have been much larger in the second subset of trials as compared to the first subset of trials. Reassuringly, these analyses revealed that the extrinsic relational Simon effect was simply unaffected by this factor, both in the error data and the response latency data, *F*s < 1. We can thus safely conclude that one can use the (extrinsic) relational Simon task to capture complex relational stimulus processing under conditions of automaticity.

## General discussion

While research on automatic stimulus processing has focused almost exclusively on the (semantic) analysis of single stimuli, increasingly more studies suggest that the realm of automatic stimulus processing extends to complex relational information [9–11, 15–17, 19–23, 26, 46, 47]. The present studies add to this line of research by showing that the (extrinsic) relational Simon task can be used to capture automatic relational stimulus processing. In each of these experiments, we observed a clear-cut (extrinsic) relational Simon effect, that is, irrespective of the type of stimulus materials used, irrespective of the similarity between the relational information that was task-relevant and the relational information that was task-irrelevant, and irrespective the complexity of the task-irrelevant relational information. We can thus conclude that the (extrinsic) relational Simon effect is a robust phenomenon that, given its procedural flexibility and simplicity, has the potential of becoming a widely applicable instrument to study (automatic) relational stimulus processing.

Importantly, there are good reasons to argue that the (extrinsic) relational Simon effect is characterized by several automaticity features [4]. Remember that the (extrinsic) relational Simon effect can occur only if participants process two (orthogonal) sources of relational stimulus information at the same time. The short response latencies, especially in in Experiment 1 and Experiment 3, thus imply that automatic relational stimulus processing is fast and efficient [4]. Moreover, the observation that participants were influenced by relational information that was task-irrelevant suggests that automatic relational stimulus processing is (at least to some degree) difficult to control [41].

Some caution is in order, however, when evaluating the extent to which participants did or did not engage in intentional processing of the relational information that was task-irrelevant. Both in Experiment 2 and 3, participants switched between two relational tasks based on the color of the to-be-compared stimuli. As a result, participants did not know which task to perform until the critical stimuli appeared on the computer screen. It is thus a possibility that (at least some) participants processed both types of relational information in an intentional manner (on at least a number of trials) to deal with this situation. Although post-hoc analyses seemed to rule out this possibility, it could be worthwhile to conduct follow-up studies examine this issue more thoroughly. For example, one might present a task cue prior to the presentation of each trial so that participants can readily ignore relational information that is task-irrelevant [41]. As an alternative approach, one could also manipulate the relative frequency of the two relational tasks so that one relational task becomes the default and the other relational task becomes the exception [44, 48]. If the extrinsic relational Simon effect were to replicate under such conditions, this would be a very strong indication that the occurrence of this effect is not critically dependent on the conscious intention to process the relational information that is task-irrelevant.

It is important to emphasize, however, that the task-switch design used in Experiments 2 and 3 is just one way to study (automatic) relational stimulus processing. In Experiment 1, participants simply performed the same perceptual matching task throughout the entire experiment and conceptual information was always task-irrelevant. Nevertheless, a clear-cut relational Simon effect emerged. This finding is a clear-cut demonstration that relational processing of task-irrelevant stimulus information can indeed take place even in the absence of the (conscious) goal to do so. Research conducted by Proctor and Healy [49] also corroborates this conclusion. They presented participants with pairs of letters strings and asked them, in one condition, to classify these pairs as ‘same’ if the strings contained the same letters, regardless of whether the letters were in the same order (and as ‘different’ if different letters were used for the two strings). Results showed that participants were slower to respond to the rearranged (but otherwise identical) letter pairs as a function of the total number of positions that letters were displaced. Such a displacement effect indicates that participants were unable to ignore the order information (which is relational by definition), despite the fact that this order information was entirely task-irrelevant (for related findings, see [50, 51]).

It must be noted, however, that both our studies and those reported by Proctor and Healy [49] are characterized by an important procedural limitation. Even though participants were not required to process the relational information that was task-irrelevant, the experimental task did require the participants to engage in relational stimulus processing. The question thus arises whether the activation of such a relational processing goal is a necessary precondition for the (extrinsic) relational Simon effect to occur (i.e., goal-dependent unintentionality, see [4]). To examine this possibility, instead of asking participants to pronounce the words ‘synonym’ and ‘antonym’ based a perceptual relation between two words (cf. Experiment 1), one may ask participants to use these relational responses based on a perceptual feature of just one of the two words. If the relational Simon effect would replicate under these conditions, this observation would provide a very strong argument in favor of the hypothesis that automatic relational stimulus processing is *not* critically dependent upon the activation of an explicit relational processing goal.

In this context, it is perhaps interesting to refer to a relatively old but ingenious sequential priming study reported by McKoon and Ratcliff [52]. In a lexical decision task, they presented participants with word pairs that were all highly associated. Crucially, each prime word was paired with two different target words to create different types of semantic relatedness. For example, in one subset of trials, synonym pairs (e.g., close–near) were contrasted with antonym pairs (e.g., close—far). Likewise, in another subset of trials, two members of the same category (e.g., car—truck) were contrasted with word pairs consisting of a category member and a category name (e.g., car—vehicle). Mckoon and Ratcliff [52] embedded these critical word pairs in a context of filler trials so that, for example, synonym/antonym pairs were tested either in a list of mostly synonym pairs or mostly antonym pairs. The results clearly showed that target responding was speeded when a pair was tested in a list of other pairs of the same relation as itself relative to a list of pairs of a different relation. For example, antonym pairs in an antonym list were associated with faster response times than did antonym pairs in a synonym list. This observation is important as it implies that participants picked up the broader relational context and applied it to the individual trials under automaticity conditions, even in the absence of an explicit relational target task. In fact, observations like those reported by McKoon and Ratcliff [52] can be taken as evidence that relational stimulus processing may very well be the rule rather than the exception (but see [53]).

This conclusion is also relevant for the field of so-called implicit measures. It is a well-known fact that the validity of self-report measures (e.g., questionnaires, interviews) can be quite low if respondents are unwilling and/or unable to self-diagnose and report the to-be-measured psychological construct [54–56]. To circumvent this problem, researchers have developed a wide range of assessment tools, often referred to as implicit measures, that allow one to capture psychological constructs under automaticity conditions [57]. Typical examples are the implicit association test [58], the evaluative priming paradigm [59, 60], the (extrinsic) affective Simon task [44], and the affect misattribution paradigm [61]. Crucially, each of these measures capitalizes on the idea that the presentation of a stimulus results in an automatic and unconditional retrieval of associated attributes from memory. In many cases, however, it is not only important to determine the extent to which two concepts (e.g., a target concept and a target attribute) are related in memory but also the precise way in which they are related [62–64]. In the context of depression, for example, it makes a huge difference to know whether an association between the self and the attribute ‘good’ reflects actual self-esteem (i.e., the belief that one is good) or ideal self-esteem (i.e., the desire to feel good about oneself, see [63–65]). To capture this type of complex relational information, (implicit) measures are needed that allow one to diagnose how individuals tend to relate different concepts. Two such measures were developed relatively recently, i.e., the implicit relational assessment procedure [66] and the relational responding task [67]. The present research adds to this literature by showing that one can also use a Simon-like procedure to capture automatic relational stimulus processing. For example, again using the same example of implicit self-esteem, one may present participants with two classes of self-descriptive adjectives. One class of adjectives, presented in a white front, consists of adjectives unrelated to self-esteem (e.g., male, female, Belgian). The other class of adjectives, presented in either a blue or a yellow front, consists of adjectives related to self-esteem (e.g., good, bad, competent). Participants can then be asked to judge whether the adjectives apply to them on the white trials and to judge the color of the adjectives on the remaining trials using the same response keys. Using such a setup, the extrinsic relational Simon effect would reflect actual self-esteem at the implicit level. The same logic can be applied to capture ideal self-esteem. Further research would be required though to ascertain whether such an approach would be sensitive and robust enough for actual use in applied settings.
